# Effects of NF-κB Inhibitor on Sepsis Depend on the Severity and Phase of the Animal Sepsis Model

**DOI:** 10.3390/jpm14060645

**Published:** 2024-06-17

**Authors:** Ye Jin Park, Jinkun Bae, Jae-Kwang Yoo, So-Hee Ahn, Seon Young Park, Yun-Seok Kim, Min Ji Lee, Seon Young Moon, Tae Nyoung Chung, Chulhee Choi, Kyuseok Kim

**Affiliations:** 1 Department of Emergency Medicine, CHA University School of Medicine, Seongnam 13497, Republic of Korea; yejin6577@naver.com (Y.J.P.); galen97@chamc.co.kr (J.B.); loupys@naver.com (Y.-S.K.); minji.lee29@gmail.com (M.J.L.); moon040659@naver.com (S.Y.M.); hendrix74@cha.ac.kr (T.N.C.); 2 Department of Emergency Medicine, CHA Bundang Medical Center, CHA University, Seongnam 13497, Republic of Korea; 3 ILIAS Biologics Inc., Daejeon 34014, Republic of Korea; jyoo@iliasbio.com (J.-K.Y.); shahn@iliasbio.com (S.-H.A.); spark@iliasbio.com (S.Y.P.)

**Keywords:** NF-κB inhibitor, hyperinflammation, rats

## Abstract

Hyperinflammation occurs in sepsis, especially in the early phase, and it could have both positive and negative effects on sepsis. Previously, we showed that a new concept of NF-κB inhibitor, exosome-based super-repressor IκBα (Exo-srIκB) delivery, has a beneficial effect on sepsis. Here, we further investigate the therapeutic effects of Exo-srIκB at different severities and phases of sepsis using an animal polymicrobial intra-abdominal infection model. We used a rat model of fecal slurry polymicrobial sepsis. First, we determined the survival effects of Exo-srIκB on sepsis according to the severity. We used two different severities of the animal sepsis model. The severe model had a mortality rate of over 50%. The mild/moderate model had a less than 30% mortality rate. Second, we administered the Exo-srIκB at various time points (1 h, 6 h, and 24 h after fecal slurry administration) to determine the therapeutic effect of Exo-srIκB at different phases of sepsis. Lastly, we determined the effects of the Exo-srIκB on cytokine production, arterial blood gas, electrolyte, and lactate. The survival gain was statistically significant in the severe sepsis model when Exo-srIκB was administered 6 h after sepsis. Interleukin 6 and interleukin-10 were significantly decreased in the kidney when administered with Exo-srIκB. The laboratory data showed that lactate, glucose, and potassium levels were significantly lowered in the NF-κB inhibitor group. In conclusion, Exo-srIκB exhibited a beneficial therapeutic effect when administered 6 h post fecal slurry administration in a severe sepsis model.

## 1. Introduction

Classically, systemic hyperinflammation, called cytokine storms, has been considered as the key pathophysiology of sepsis, especially in the early phase [1,2,3]. Consequently, drugs targeting hyperinflammation have been developed in preclinical studies and tested in clinical situations, but all failed [3]. Only steroids have limited value in sepsis. The surviving sepsis guidelines recommend steroids only in septic shock and as an ongoing requirement for vasopressor therapy [4]. They do not recommend steroids in sepsis without shock. However, dexamethasone, which reduces inflammation, is known to be an effective treatment option for COVID-19. COVID-19 is also sepsis of viral infection. This complex result could be partly due to the fact that therapeutic approaches can differ depending on sepsis severity [5]. We also showed that dexamethasone could be beneficial in sepsis if used early in the severe sepsis model, and with this preclinical result, we are conducting clinical trials in sepsis using dexamethasone [6,7].

**Figure 1:**
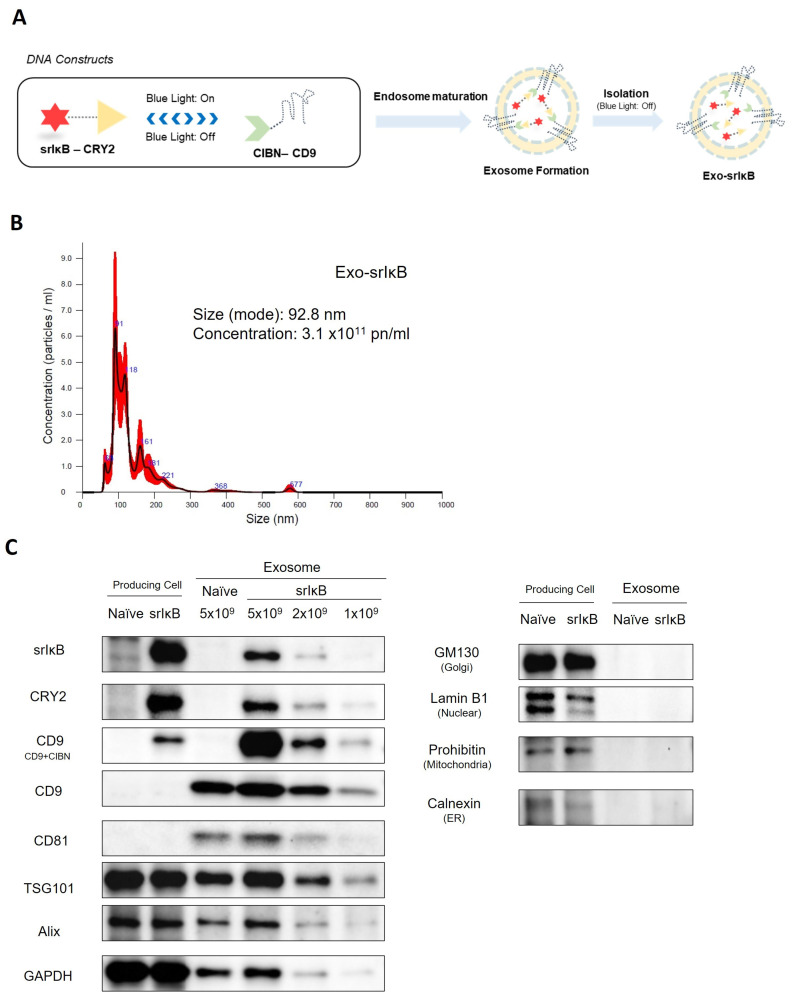
Characterization of Exo-srIκB. (**A**) Schematic diagram of DNA constructs used for the production of super-repressor IκB-loaded exosome (Exo-srIκB) and biogenesis of cargo protein-carrying exosome. For active loading of srIkB into the exosomes, we engineered a human embryonic kidney (HEK) 293 cell line that stably expresses two recombinant proteins, CIBN-CD9 and srIκB-CRY2. Upon blue light illumination, light-inducible protein interaction between CRY2 and CIBN can recruit srIkB-CRY2 into the lumen of the exosomes via natural exosome biogenesis. Then, srIkB-CRY2 can be released as free forms in the lumen of exosomes by removing light stimulation. (**B**) Concentration and size distribution of Exo-srIκB were determined by a Nanosight (NS300) instrument(Malvern Panalytical, Malvern, UK). (**C**) Target proteins (srIκB, CRY2, and CD9), exosome positive markers (CD9, CD81, TSG101, Alix, and GAPDH), and cell organelle markers (exosome negative marker; GM130, Lamin B1, Prohibitin, and Calnexin) expressed in the cells and loaded into cell-derived exosomes were analyzed by immunoblotting. Naïve cells and Exo-naïve were used as negative controls of Exo-srIκB.

NF-κB is the central player in hyperinflammation in sepsis, and more than 700 types of NF-κB inhibitors were developed and tested. However, most were small chemicals with low specificity and significant off-target effects, so they failed in clinical trials due to side effects. Super-repressor IkB, on the other hand, has very high specificity, but its size reaches 50 kDa, so effective intracellular delivery is required. Therefore, we applied previously developed exosome-based intracellular polymer protein delivery technology. Previously, we found exosomes as a therapeutic carrier to deliver super-repressor IκB (srIκB), Exo-srIκB, to be beneficial in preclinical mouse sepsis models (Figure 1). [8]. However, we did not test whether the therapeutic effects of anti-inflammatory exosomes might be dependent on time points of administration and severity of sepsis. Here, we investigate the effects of an exosome-based NF-κB inhibitor, Exo-srIκB, on sepsis depending on the severity and phase of sepsis.

## 2. Materials and Methods

### 2.1. Exosomes Production

The exosome production process was previously described [8,9]. In brief, Exo-srIκB producing cells were incubated for 4 days in a wave culture system. For batch consistency and scalable production, we chose a transformed cell line, the HEK293F (Expi293F) cell line, a variant of HEK293T cells. The HEK293F cell line, adapted for high-density, serum-free suspension culture, is ideal for large-scale biopharmaceutical production, including therapeutic exosomes. We confirmed that HEK293F-derived exosomes are quite safe in preclinical toxicity studies and phase 1a clinical studies. The producing cells were exposed to blue light illumination for target protein loading. After that, the culture medium was harvested and centrifuged. Next, the exosome was purified through ultrafiltration and diafiltration and was purified through anionic and multi-modal resin chromatography. Finally, a formulation and sterilization filter process was performed.

### 2.2. Nanoparticle Tracking Analysis

Particle number and size distribution of EVs were assessed utilizing nanoparticle tracking analysis (NTA) with the NS300 instrument (Malvern Panalytical, a Spectris company, UK). Consistent with the manufacturer’s guidelines, the samples underwent analysis under steady flow conditions at a temperature of 25 °C, with a camera level of 15 and a detection threshold of 3. The concentration of EVs was measured by sample dilution ranging from 1:100 to 1:10,000, with particle counts in the range of 20 to 100 per frame.

### 2.3. Western Blotting

To initiate Western blotting analysis, both producing cells and exosomes were lysed. Producing cells were lysed in RIPA buffer containing Halt™ Protease and Phosphatase Inhibitor Cocktail (100X) (Thermo Fisher Scientific, Waltham, MA, USA), while exosomes were lysed in 4X Laemmli sample buffer (Bio-RAD, Hercules, CA, USA). Samples were separated on a 10% SDS/polyacrylamide gel electrophoresis (PAGE) gel and then transferred onto nitrocellulose membranes. These membranes were then incubated with primary antibodies specific to srIκB, CRY2 (customized antibody, Abclon, Seoul, Republic of Korea), CD9, CD81 (SBI, Tokyo, Japan), TSG101, Alix, GM130, calnexin (Abcam, Cambridge, UK), Lamin B1, Glyceraldehyde-3-phosphate dehydrogenase (GAPDH) (Santa Cruz Biotechnology, Dallas, TX, USA), and Prohibitin (NOVUSBIO, Centennial, CO, USA) at 4 °C overnight. Following the primary antibody incubation, specific secondary antibodies were applied, and the blots were developed using Clarity and Clarity Max ECL Western Blotting Substrates. The resulting blots were imaged using a ChemiDoc imager (Bio-Rad, Hercules, CA, USA).

### 2.4. In Vivo Sepsis Model Induction

This research received approval from the Institutional Animal Care and Use Committee (IACUC) of our institute (Approval No. IACUC-220066), conforming to the National Institutes of Health guidelines. Male Sprague–Dawley rats, weighing between 280 and 320 g, were purchased from KOATECH Inc. (Pyeongtaek-si, Republic of Korea) for the experimental procedures. These animals were then acclimatized to a specific pathogen-free environment, with regulated temperature (20–24 °C), humidity (40–60%), and alternating 12 h light/dark cycles. Access to standard alimentation and water was provided ad libitum for a duration of seven days prior to the commencement of the experimental protocols.

Our methodology included the application of a polymicrobial intra-abdominal sepsis model, adjusted for body weight, as delineated in preceding studies [10,11]. While the cecal ligation and puncture (CLP) model is commonly employed in sepsis research, it has limitations, including variable fecal characteristics among subjects, inconsistency in puncture sites, and variations in applied pressure during the procedure, potentially leading to disparate outcomes ranging from fulminant sepsis to intra-abdominal abscess. Consequently, we selected the cecal slurry method [12]. This technique used donor rats. They were anesthetized with intramuscular Zoletil (50 mg/kg) and xylazine (10 mg/kg). A subsequent midline laparotomy exposed the cecum, from which fecal contents were collected through a 0.5 cm incision made on the antimesenteric border. The donor rats were then euthanized. The collected fecal material was quantified, subsequently diluted with a 5% dextrose saline solution in a 1:3 ratio, and prepared into a fecal slurry.

For the induction of sepsis, recipient rats underwent the same anesthetic protocol prior to a 0.5 cm midline laparotomy, where the homogenized fecal slurry was introduced into the peritoneal cavity. Preceding intraperitoneal administration, slurry suspensions were standardized via vortexing. The volume administered was meticulously calculated relative to the body weight of the recipient rat. Post-procedure, the animals received fluid resuscitation subcutaneously (30 mL/kg using 5% dextrose saline), accompanied by subcutaneous injections of imipenem at a dosage of 25 mg/kg twice daily for a period of 48 h. Analgesics were not used in this protocol. Following these procedures, stratified randomization based on body weight was conducted by a research assistant.

**Experiment 1:** Survival study according to the severity and phase of sepsis.

We used two severity models: severe or mild/moderate sepsis. The severity of sepsis was defined based on the mortality of the control group. Mortality higher than or equal to 50% is defined as severe, and less than 30% as a mild/moderate sepsis model. We adjusted the volume of fecal slurry according to the severity of sepsis. We also performed a survival study according to the phase of sepsis. We defined 1 h, 6 h, and 24 h after induction of sepsis as hyperacute phase, acute phase, and late phase, respectively. At each time point, we administered 4.5 × 10^10^ particles of Exo-srIκB to the rat via the tail vein. The exosome dosage was determined based on the effective doses identified in our previous studies with different disease models [8]. As a control, an equivalent volume of PBS was injected in the same manner. Rats were randomly allocated to the following groups: the Exo-srIκB group and the vehicle (Control) group. Survival was monitored every 12 h for 14 days.

**Experiment 2:** Blood chemistry study: In this study, Exo-srIκB was intravenously administered to rats through the tail vein 6 h post the initiation of severe sepsis. Blood sampling was performed 24 h after fecal slurry administration. The study groups were stratified into three: a sepsis-only group (Control), a sepsis group receiving Exo-srIκB treatment (Exo-srIκB), and a non-septic sham group (Sham). Plasma lactate, glucose levels, arterial blood gas values, and electrolyte concentrations were quantitatively assessed.

In all experiments, sepsis was confirmed through the observation of reduced motor activity, lethargy, shivering, and piloerection [13]. Given the study design, potential confounders were deemed unlikely to influence the results and were therefore not controlled for.

### 2.5. Kidney Cytokines (Enzyme-Linked Immunosorbent Assays (ELISA))

A total of 22 kidney samples were gathered from rats. The rats were divided into three groups: Sham, Control, and Exo-srIκB. Samples were homogenized in DPBS, and supernatant was used for ELISA. Cytokines were measured by Rat IL-10 ELISA Kit (ab214566, Abcam, Cambridge, UK) and Rat IL-6 Quantikine ELISA Kit (R6000B, R&D SYSTEMS, Minneapolis, MN, USA) according to the manufacturer’s instructions.

### 2.6. Plasma Lactate, Glucose, Arterial Blood Gas Analysis, and Electrolytes

Plasma lactate, glucose, arterial blood gas analysis, and electrolytes were performed using arterial blood samples via the abdominal artery (ABL90 Flex Plus, Radiometer, DK). The blood sample was collected using a heparinized syringe.

### 2.7. Statistical Analysis

Data were expressed as mean ± standard deviation (SD). The normality for the distribution of variables was verified by the Shapiro–Wilk test. A one-way ANOVA with Bonferroni post hoc tests was used to compare means in the normally distributed data. If the distributions were not normal, the data were analyzed using the Kruskal–Wallis test. Survival probabilities were calculated using the Kaplan–Meier method. All *p*-values < 0.05 were considered significant. Statistical analysis was performed using EZR software, version 1.54 (Saitama Medical Center, Jichi Medical University, Saitama, Japan).

## 3. Results

### 3.1. Survival Study According to the Exo-srIκB Depending on Severity of Sepsis and Phase of Sepsis

In the mild/moderate sepsis model, administration of Exo-srIκB on hyperacute and acute (1 and 6 h after fecal slurry administration) and late (24 h after fecal slurry administration) phase of sepsis had no effects on survival with a tendency to decrease the survival rate.

**Figure 2:**
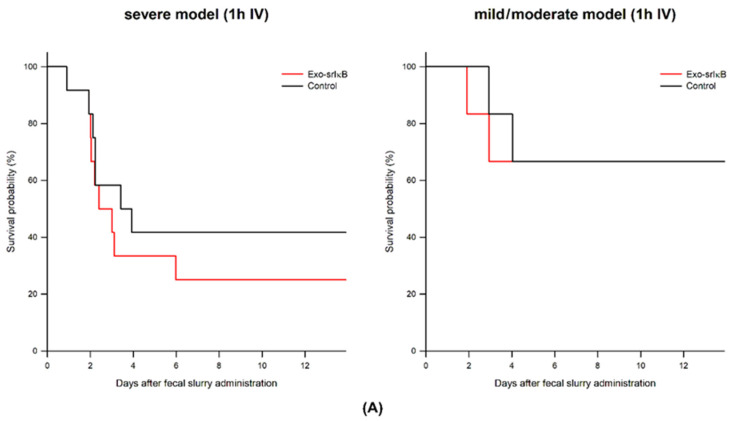

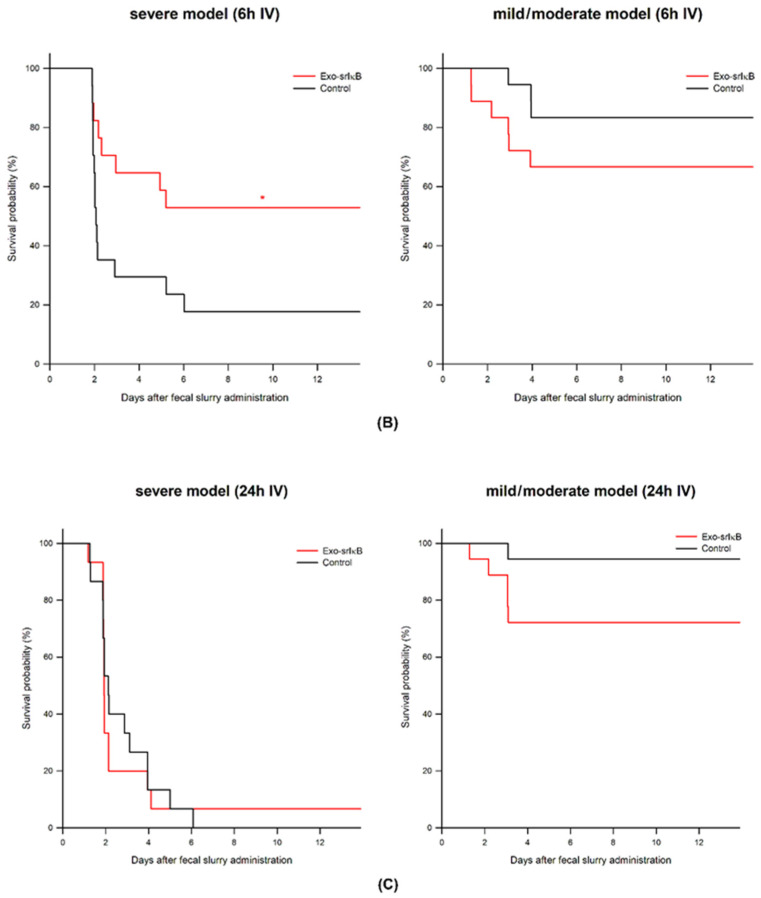
Survival effects of Exo-srIκB on the polymicrobial sepsis model according to the severity of sepsis and phase of sepsis. (**A**) Exo-srIκB was administered 1 h after severe and mild/moderate sepsis. (Control and Exo-srIκB, *n* = 18 per group) (**B**) Exo-srIκB was administered 6 h after severe and mild/moderate sepsis. (Control and Exo-srIκB, *n* = 17~18 per group) * *p* < 0.05 compared with Control (**C**) Exo-srIκB was administered 24h after severe and mild/moderate sepsis. (Control and Exo-srIκB, *n* = 15~18 per group).

In the severe sepsis model, administration of the Exo-srIκB group in the acute phase (6 h after fecal slurry administration) showed an improved survival rate (17.7% vs. 52.9%); however, there was no survival difference between the Control and Exo-srIκB group when it was administrated at 1 h or 24 h after fecal slurry administration (Figure 2).

### 3.2. Kidney Cytokines

**Figure 3:**
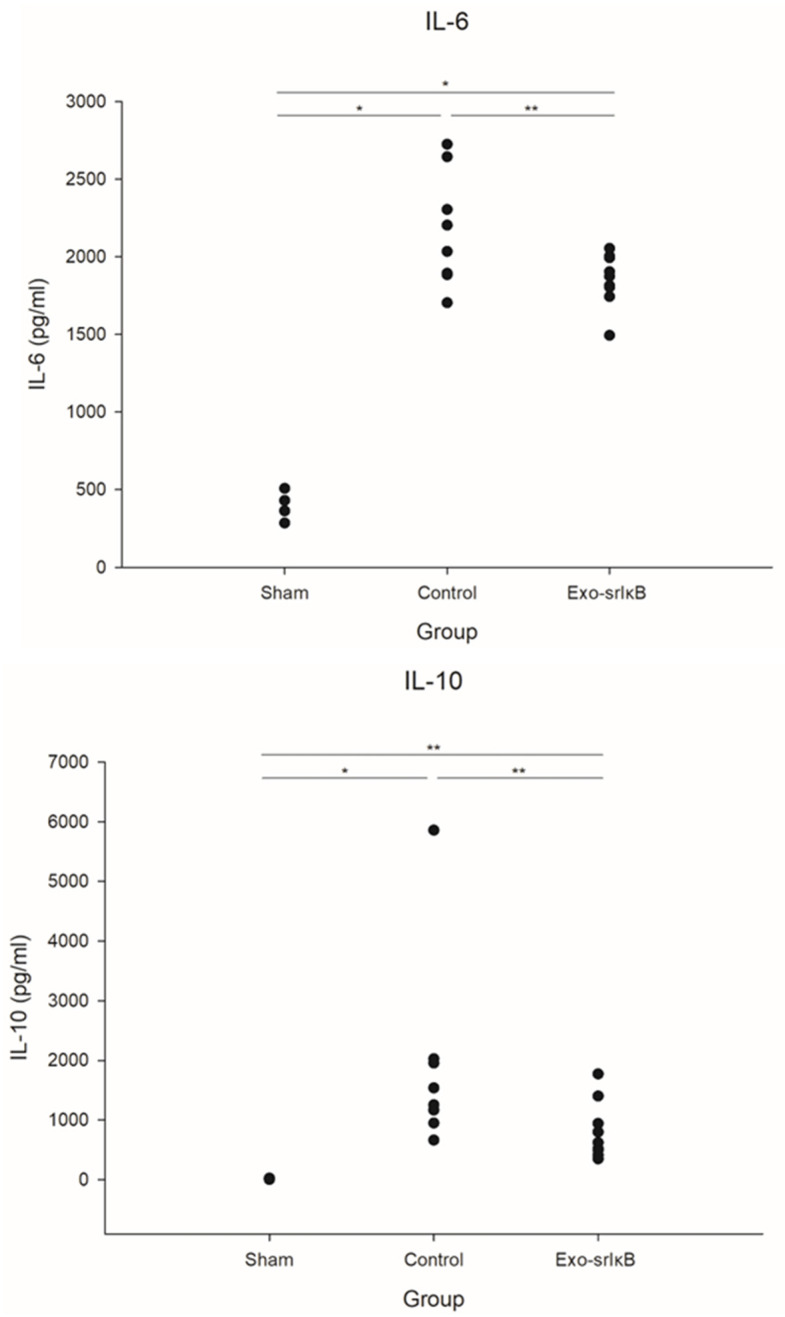
Kidney cytokines in a polymicrobial severe sepsis model. (**A**) IL = 6, (B) IL-10 (Sham *n* = 5; Control *n* = 8, and Exo-srIκB, *n* = 9) * *p* < 0.05 compared with Sham using Kruskal–Wallis or ANOVA; ** *p* < 0.05 compared with Exo-srIκB using ANOVA or *t*-test.

The cytokine production in sepsis induction is shown in Figure 3. Administration of Exo-srIκB to the subjects resulted in modified cytokine profiles. There was a significant reduction in the concentrations of IL-6 (2174 ± 367 pg/mL vs. 1854 ± 170 pg/mL) and IL-10 (1927 ± 1655 pg/mL vs. 813 ± 484 pg/mL) in the Exo-srIκB-treated group relative to the Control group, indicating a significant attenuation of the cytokine response.

### 3.3. Plasma Lactate, Glucose, Arterial Blood Gas Analysis, and Electrolytes

**Figure 4:**
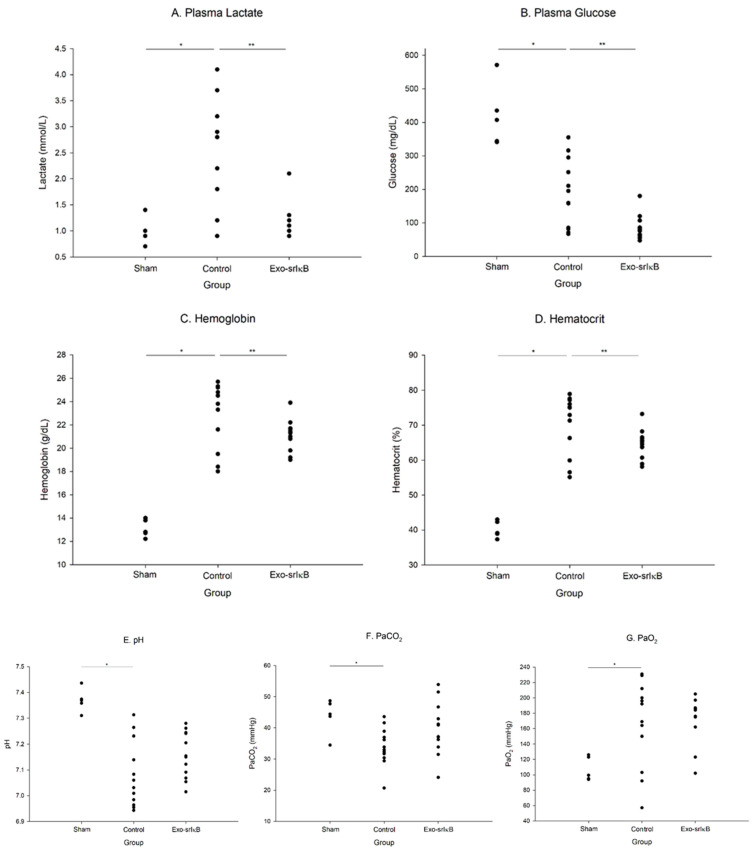

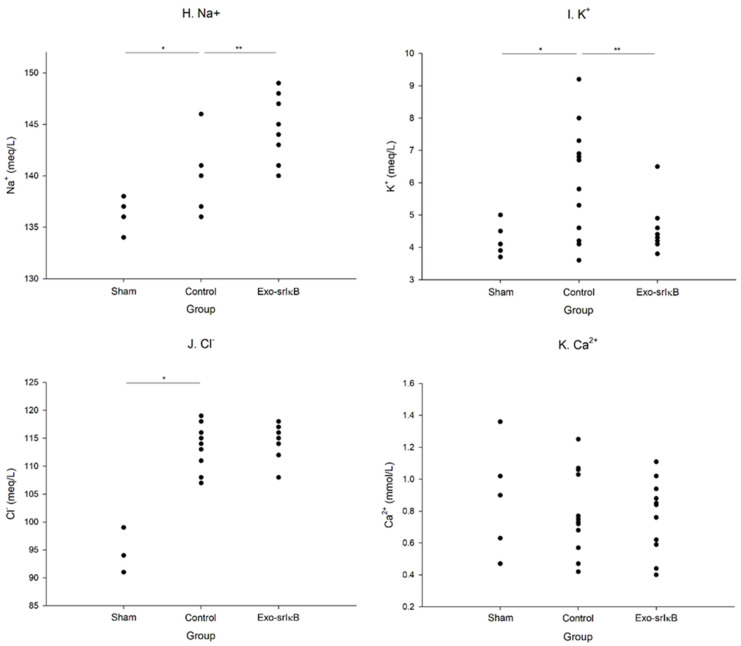
Plasma lactate, glucose, arterial blood gas analysis, and electrolytes in a polymicrobial severe sepsis model. (**A**) Plasma Lactate, (**B**) Plasma Glucose, (**C**) Hemoglobin, (**D**) Hematocrit, (**E**) pH, (**F**) PaCO_2_, (**G**) PaO_2_, (**H**) Na^+^, (**I**) K^+^, (**J**) Cl^-^, (**K**) Ca^2+^. (Sham *n* = 5; Control and Exo-srIκB, *n* = 11 to 12 per group) * *p* < 0.05 compared with Sham using Kruskal–Wallis or ANOVA; ** *p* < 0.05 compared with Exo-srIκB using ANOVA or *t*-test.

There were significant reductions in the level of lactate, glucose, potassium, hemoglobin, hematocrit, and increment sodium levels when the Exo-srIκB was administered (Figure 4).

## 4. Discussion

We showed that Exo-srIκB had beneficial effects on sepsis only in the severe sepsis model when administered 6 h after fecal slurry administration.

In the hyperacute phase of sepsis (in this study, 1 h after fecal slurry administration), the inflammation reaction to the pathogen might be critical, and with this immune response, pathogens could be cleared. When this reaction is mitigated, the pathogen could survive and destroy our own body. In this study, the NF-κB inhibitor administered 1 h after fecal slurry administration had no survival gain, and it even seemed to be harmful. This supports the above hypothesis. However, if this immune reaction is too high in the later phase of sepsis after pathogens are eliminated, it could be self-harmed, which is called a cytokine storm. Cytokine storms should be blocked to some extent, and we think the NF-κB inhibitor 6 h after severe sepsis in this study represents that time window. We also confirmed no beneficial effect of NF-κB inhibitor on sepsis 24 h after fecal slurry administration, which had no survival effects. This time point could be too late to block cytokine storms.

Exo-srIκB had no effects on the mild/moderate sepsis model at every time point. Cytokine storms have been known to occur in severe sepsis, and NF-κB inhibitors without cytokine storms might have no effect. On the contrary, the survival rate had a tendency to decrease in the Exo-srIκB group when administered in mild/moderate sepsis. This result is reasonable considering the inhibitory effects of the NF-κB inhibitor on phagocytosis [14]. Supporting this concept, dexamethasone, another anti-inflammatory drug, has been effective only in moderate to severe COVID-19, such as hypoxia and high CRP levels [15]. It has also been reported that dexamethasone might be harmful in mild COVID-19 patients [15,16].

Administration of Exo-srIκB led to a significant reduction in plasma lactate levels. Lactate serves as a crucial indicator of sepsis severity, thus indicating that Exo-srIκB mitigated the severity of sepsis. Elevated hemoglobin/hematocrit levels in sepsis may indicate hemoconcentration resulting from fluid deficiency in sepsis. Exo-srIκB dampened inflammation, consequently reducing fluid loss.

As correlated with previous studies with dexamethasone in sepsis [7], this study suggests that immunosuppressants, i.e., anti-hyperinflammatory drugs, should be used with caution in sepsis. It might be a double-edged sword, which has both beneficial and harmful effects, depending on the phase and severity of sepsis. Determining the optimal timing for administering this pharmaceutical agent in actual clinical scenarios presents a significant challenge. Take, for instance, the context of septic patients who present to the emergency department (ED) and receive treatments including fluid resuscitation, antibiotic therapy, and occasionally vasopressors or corticosteroids; the subsequent initiation of NF-κB inhibitor therapy upon their admission to the intensive care unit (ICU) could be considered delayed. Consequently, an interim period that spans from the patient’s arrival at the ED to their transfer to the ICU may represent a more suitable therapeutic window. To navigate this complexity, the development and utilization of a real-time biomarker would be indispensable.

This study has limitations to consider. We administered the drug at various time points, and we only performed a survival study without investigating the effects on cytokine and blood biochemistry. The differences in cytokines and blood biochemistry at these time points might establish a correlation between the blood biochemistry/cytokine level and the efficacy of Exo-srIκB.

## 5. Conclusions

A new concept of NF-κB inhibitor, Exo-srIκB, had beneficial effects only in the acute phase of severe sepsis in a polymicrobial sepsis model.

## Data Availability

The datasets generated and analyzed during the current study are available from the corresponding author upon reasonable request.
